# Recent Advances in Developing High‐Performance Anode for Potassium‐Ion Batteries based on Nitrogen‐Doped Carbon Materials

**DOI:** 10.1002/smll.202406630

**Published:** 2024-10-07

**Authors:** Yonghuan Fu, Yulian Dong, Yonglong Shen, Huaping Zhao, Guosheng Shao, Yong Lei

**Affiliations:** ^1^ Fachgebiet Angewandte Nanophysik Institut für Physik & IMN MacroNano Technische Universität Ilmenau 98693 Ilmenau Germany; ^2^ School of Materials Science and Engineering Zhengzhou University Zhengzhou 450001 China

**Keywords:** anode materials, edge‐nitrogen doping, electrochemical performance, nitrogen‐doped carbon, potassium‐ion batteries

## Abstract

Owing to the low potential (vs K/K^+^), good cycling stability, and sustainability, carbon‐based materials stand out as one of the optimal anode materials for potassium‐ion batteries (PIBs). However, achieving high‐rate performance and excellent capacity with the current carbon‐based materials is challenging because of the sluggish reaction kinetics and the low capacity of carbon‐based anodes. The doping of nitrogen proves to be an effective way to improve the rate performance and capacity of carbon‐based materials as PIB anode. However, a review article is lacking in systematically summarizing the features and functions of nitrogen doping types. In this sense, it is necessary to provide a fundamental understanding of doped nitrogen types in nitrogen‐doped(N‐doped) carbon materials. The types, functions, and applications of nitrogen‐doped carbon‐based materials are overviewed in this review. Then, the recent advances in the synthesis, properties, and applications of N‐doped carbon as both active and modification materials for PIBs anode are summarized. Finally, doped nitrogen's main features and functions are concluded, and critical perspectives for future research in this field are outlined.

## Graphical abstract

While nitrogen doping is a common technique for carbon material modification, the features and functions of doped nitrogen types in nitrogen‐doped (N‐doped) carbon materials remain underexplored. This review addresses this gap by offering an overview of doped nitrogen's main features and functions in enhancing potassium ion batteries (PIBs) rate performance and capacity. It covers recent progress and prospects related to PIBs.

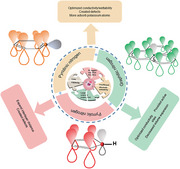

## 1 Introduction

**Figure 1:**
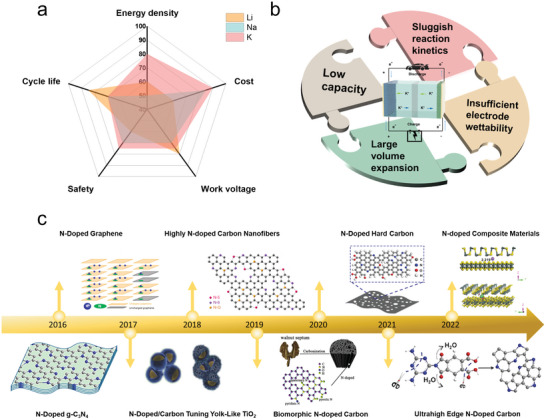
a) Key battery parameters compared between PIBs, LIBs, and SIBs. b) The challenges of anode material for PIBs. c) The current progress of the development of N‐doped carbon materials. Nitrogen‐Doped Graphene. Reproduced with permission.^[^ ^37^ ^]^ Copyright 2016, American Chemical Society. N‐Doped g‐C_3_N_4_. Reproduced with permission.^[^ ^30^ ^]^ Copyright 2017, American Chemical Society. N‐Doped/Carbon Tuning Yolk‐Like TiO_2_. Reproduced with permission.^[^ ^38^ ^]^ Copyright 2017, Willey‐VCH. Highly N‐doped Carbon Nanofibers. Reproduced with permission.^[^ ^39^ ^]^ Copyright 2018, Springer. Biomorphic N‐doped Carbon. Reproduced with permission.^[^ ^40^ ^]^ Copyright 2019, Elsevier. N‐Doped Hard Carbon. Reproduced with permission.^[^ ^41^ ^]^ Copyright 2020, Willey‐VCH. Ultrahigh Edge N‐Doped Carbon. Reproduced with permission.^[^ ^42^ ^]^ Copyright 2021, Willey‐VCH. N‐doped Composite Material. Reproduced with permission.^[^ ^43^ ^]^ Copyright 2022, Elsevier.

Battery technology is essential for large‐scale renewable energy storage toward addressing the global overdependence on fossil fuels as well as the resulting greenhouse effect and environmental problems.^[^ ^1^, ^2^, ^3^ ^]^ In this respect, lithium‐ion batteries (LIBs) are promising owing to their high energy density.^[^ ^1^, ^4^, ^5^, ^6^, ^7^ ^]^ Still, the large‐scale applications of LIBs for renewable energy storage are restricted to the high‐cost and shortage of lithium resources. Sodium‐ion batteries (SIBs),^[^ ^8^, ^9^, ^10^ ^]^ while potentially low‐cost attributing to the abundant sodium resources, are limited to their low energy density.^[^ ^10^, ^11^, ^12^ ^]^ Therefore, potassium‐ion batteries (PIBs) turn out to be the trade‐off between LIBs and SIBs, making PIBs ideal for large‐scale renewable energy storage applications. First of all, the standard reduction potential of K^+^/K is −2.93 V (vs standard hydrogen potential [SHE]) is close to that of Li^+^/Li (−3.04 V vs SHE), which enables PIBs to have a similar energy density as LIBs.^[^ ^2^, ^13^, ^14^ ^]^ Moreover, the redox potential of K^+^/K (−2.88 V vs saturated calomel electrode [SCE]) in propylene carbonate (PC) solvent is lower than those of Li^+^/Li (−2.79 V vs SCE) and Na^+^/Na (−2.56 V vs SCE),^[^ ^3^, ^8^, ^15^, ^16^, ^17^ ^]^ and thus PIBs can have a higher energy density than those of LIBs and SIBs in the case of using nonaqueous electrolytes (**Figure** **1**a). Second, potassium is much more abundant in Earth's crust than lithium, making PIBs low‐cost.^[^ ^2^, ^18^, ^19^, ^20^ ^]^ In addition, since potassium cannot alloy with aluminum at low potentials, the aluminum foil, instead of the expensive copper foil for anodes in LIBs, can be employed as the current collector for anodes in PIBs, which can further reduce the price of PIBs.^[^ ^21^, ^22^, ^23^ ^]^ Accordingly, PIBs have attracted great attention in recent years, and significant research progress has been made in developing high‐performance PIBs.

However, one of the critical issues in developing cost‐effective PIBs is the lack of optimal anode materials. Many anode materials for PIBs have been explored so far, but most of them still suffer from one or more shortcomings, including sluggish reaction kinetics, large volume expansion, insufficient electrode wettability, and low capacity (Figure 1b).^[^ ^3^, ^18^, ^21^, ^22^ ^]^ Till now, carbon‐based materials are one of the most studied anode materials for PIBs, for their low potential (vs K/K^+^), good conductivity, and sustainability.^[^ ^14^, ^24^, ^25^, ^26^, ^27^ ^]^ Nevertheless, due to the big ion radius of potassium, the insertion of potassium ions (K^+^) into carbon anodes is difficult and meanwhile leads to a huge volume expansion of carbon anode, which subsequently results in poor rate performance and low cycling stability of carbon anodes.^[^ ^26^, ^27^ ^]^ Besides, the capacity of carbon anodes is also not satisfied enough. Heteroatom doping is one of the most effective strategies to improve the anode performance of carbon‐based materials. In particular, doping of nitrogen has been proven as an effective way to improve the performance of carbon‐based materials as PIBs anode. Nitrogen‐doped carbon (N‐doped) materials have experienced a long history of development. The current progress of the development of N‐doped carbon materials is shown in Figure 1c. Nitrogen‐doped carbon materials usually have improved conductivity, increased interlayer distance, and additional K^+^ absorption sites, which in turn are beneficial for improving rate performance, long‐cycle stability, and capacity.^[^ ^28^, ^29^, ^30^ ^]^ Not only as advanced anode materials, N‐doped carbon materials can also be composited with other non‐carbonaceous PIBs anode materials, e.g., forming a homogeneous carbon layer on the surface of the host non‐carbonaceous anode materials.^[^ ^31^, ^32^, ^33^ ^]^ By taking advantage of the high conductivity and excellent mechanical stability of N‐doped carbon materials, the rate capability and cycle life of the host non‐carbonaceous anode materials can be significantly improved. Nonetheless, some key issues need to be resolved for developing high‐performance PIBs anode based on N‐doped carbon materials, such as low content of nitrogen atoms in the carbon matrix, difficult control in the ratios of different types of nitrogen, and low initial Coulombic efficiency (ICE).^[^ ^34^, ^35^, ^36^ ^]^

In this review, we summarize the recent advances in the synthesis, properties, and applications of N‐doped carbon as both active materials and modification materials for PIBs anode. We first provide a fundamental understanding of three different types of doped nitrogen in N‐doped carbon materials, including their main features and functions. Then, we systematically review the synthesis and applications of N‐doped carbon materials as PIBs anode. After that, we summarize the recent progress in applying N‐doped carbon as modification materials to optimize the electrochemical performance of non‐carbonaceous PIBs anode. Finally, we also highlight the remaining challenges and propose possible solutions for the future development of high‐performance PIBs anode based on N‐doped carbon materials.

## 2 Nitrogen in N‐Doped Carbon Materials: Types and Functions

**Figure 2:**
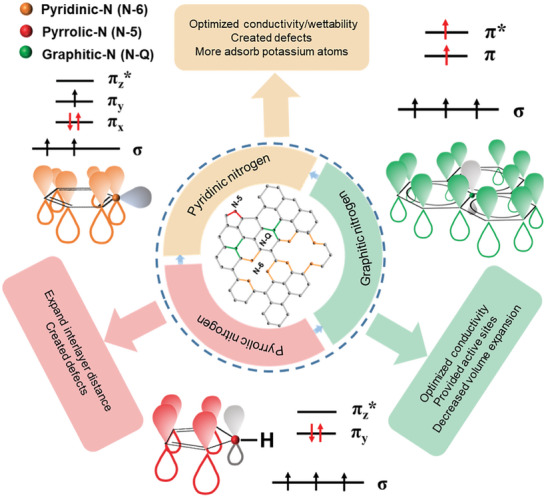
Recent research summarizes the functions of N‐doped carbon materials.

Introducing heteroatoms into the graphitic structure is an efficient method to optimize the physicochemical properties of carbon materials. The nitrogen atom is one of the typical representatives of heteroatoms that can be feasibly doped into carbon materials. The atomic radius of the nitrogen atom (0.74 Å) is close to that of the carbon atom (0.77 Å), thus significant lattice mismatches can be avoided when replacing carbon atoms in the graphitic framework with nitrogen atoms. However, the electronegativity of nitrogen (3.04) differs from that of carbon (2.55), which perturbates the electron distribution of the graphitic structure. Moreover, the degree of this perturbation also depends on the configuration of doped nitrogen in the matrix of a graphitic carbon framework. Since Masayuki Kawaguchi began the research of N‐doped carbon materials in 1992,^[^ ^37^ ^]^ N‐doped carbon materials have received considerable attention due to the potential advantages of the introduction of N atoms. Generally, three main types of nitrogen atoms are present in N‐doped carbon materials: pyridinic‐N (N‐6), pyrrolic‐N (N‐5), and graphitic‐N (or quaternary‐N, N‐Q), as illustrated in **Figure** **2**.^[^ ^44^, ^45^, ^46^ ^]^

1. Pyridinic‐N (N‐6) refers to the nitrogen atom that replaces one carbon atom in a hexagonal carbon lattice to form a pyridine‐like structure. In such a configuration, pyridinic‐N is sp^2^‐hybridized: two valence electrons of the nitrogen atom form two σ bonds with two carbon atoms, while three remaining valence electrons form one π state and a lone pair occupying an sp^2^ orbital.^[^ ^46^, ^47^, ^48^ ^]^ Due to the unpaired *p* electrons, the pyridinic‐N can accept electrons from the adjacent carbon atoms, thus causing charge redistribution in the graphitic carbon framework.^[^ ^49^ ^]^ At the same time, extra *p* electrons from pyridinic‐N lead to an increase in the electron cloud density of the overall π system, thereby improving the electrical conductivity of carbon materials. In addition, pyridinic‐N is normally located at the edges or defects of the graphitic carbon framework, which can create additional active sites for (electro)chemical reactions of carbon materials.^[^ ^50^ ^]^ While pyridinic‐N is known to improve electronic properties by introducing additional active sites, it also creates defects in the carbon matrix. Moderate amounts of pyridinic‐N can enhance electrical conductivity; however, an excessively high concentration can lead to significant structural disruption. The defects created by rich pyridinic‐N reduce the effective pathways for electron transport, thereby decreasing the overall conductivity of the material. Thus, while pyridinic‐N can be beneficial in moderate quantities, its excessive presence is detrimental to the carbon‐based material's conductivity.
2. Pyrrolic‐N (N‐5) is the hydrogen‐terminated nitrogen atom bonded to two carbon atoms in the pentagonal ring to form a pyrrole‐like structure.^[^ ^7^, ^51^, ^52^ ^]^ Unlike pyridinic‐N, pyrrolic‐N is sp^3^‐hybridized: three valence electrons of the nitrogen atom form three σ bonds, and two valence electrons form a *p* orbital lone pair. More importantly, the *p* orbital lone pair in pyrrolic‐N is perpendicular to the graphene plane, while the lone pair in pyridinic‐N is parallel to the graphene plane.^[^ ^53^ ^]^ As a result, the graphene interlayer distance in carbon materials can be significantly expanded due to the electrostatic repulsion of pyrrolic‐N.^[^ ^29^, ^48^, ^52^, ^54^ ^]^ The pyridinic‐N is usually located on the edges of the graphitic carbon framework, leading to the creation of more defects in the graphene plane. These defects serve as active sites for electrochemical reactions. Nitrogen doping, particularly with pyridinic‐N and pyrrolic‐N, often results in the formation of defects, surface functional groups, and an increased specific surface area. These characteristics, while enhancing K^+^ adsorption capacity, also contribute to a higher irreversible capacity during initial cycling. The increased surface area and defects can lead to significant electrolyte decomposition and consumption during the formation of the solid electrolyte interphase (SEI) film, resulting in a low initial Coulombic efficiency (ICE). Therefore, while nitrogen doping can enhance capacity, it simultaneously reduces ICE due to increased irreversible side reactions.
3. Graphitic‐N (N‐Q) refers to nitrogen atom that substitutes one carbon atom in the hexagonal ring. In the graphitic‐N configuration, the nitrogen atom is sp^2^‐hybridized: three valence electrons of the nitrogen atom form three σ bonds with three carbon atoms, and two valence electrons form π and π^*^ state. Different from p‐type behavior for pyridinic‐N and pyrrolic‐N due to defects that cause the Fermi level to move into the valence band, first‐principles calculations found that the introduction of graphitic‐N causes the Fermi level to shift into the conduction band, so the graphitic‐N makes the N‐doped carbon materials have n‐type behavior.^[^ ^55^ ^]^ Thus, graphitic‐N, due to its abundance of electrons, can significantly enhance the electrical conductivity of carbon‐based materials. Additionally, the graphitic nitrogen's presence could lower the adsorption energy barrier, making the potassium ions' interaction with the carbon surface more energetically favorable. Graphitic‐N differs from pyridinic‐N and pyrrolic‐N in that it does not create defects in the carbon matrix and, due to its electron‐rich nature, significantly enhances the electrical conductivity of carbon‐based materials. However, graphitic‐N exhibits lower reactivity, which can limit its effectiveness in applications where active sites for K^+^ adsorption are crucial. Although graphitic‐N offers advantages in maintaining structural stability during cycling, its lower reactivity compared to other nitrogen forms may restrict its utility in enhancing electrochemical performance where a high density of active sites is required.

As mentioned above, different types of nitrogen incorporation into carbon materials will have different effects on the structural and electronic properties, and consequently the electrochemical performance of the obtained N‐doped carbon materials as anode for PIBs. First of all, the electrical conductivity could be increased through the nitrogen introduction. On the one hand, the increased electrical conductivity will improve the rate performance of N‐doped carbon materials as PIBs anode. On the other hand, highly‐conductive N‐doped carbon materials can be composited with non‐carbonaceous PIBs anode materials to optimize the rate performance of the host non‐carbonaceous PIBs anode materials. Second, doping with pyridinic‐N and pyrrolic‐N can generate more structural defects in N‐doped carbon materials. These defects can serve as active sites for storing potassium ions via the surface‐induced capacitive process, thereby improving the capacity of N‐doped carbon materials as anode for PIBs. Third, pyrrolic‐N can expand the graphene interlayer distance of carbon materials to a larger extent. The enlarged graphene interlayer spacing will facilitate the potassium‐ion insertion/extraction reactions during the fast charging‐discharging process. At the same time, the enlarged graphene interlayer spacing could buffer the volume expansion during the insertion and extraction of potassium ions, thus enhancing the long‐term cycle stability. Fourthly, the defects created by the introduction of pyridinic‐N and pyrrolic‐N are able to optimize the surface wettability, which also benefits improving the electrochemical performance of N‐doped carbon materials as anode for PIBs. Therefore, N‐doped carbon materials show great potential in developing high‐performance (i.e., high capacity, excellent rate capability, and long‐term stability) anode for PIBs.

## 3 Recent Progress in Nitrogen‐Doped Carbon Materials as Anode for PIBs

Carbon materials are one of the most commonly used PIBs anode materials. However, carbon materials have poor cycle stability due to the huge volume expansion caused by the insertion of potassium ions (K^+^). Generally, carbon anode materials are classified into three main types: graphitic carbon, hard carbon, and soft carbon. Nitrogen‐doping is one of the most effective strategies to improve the electrochemical performance of carbon anode materials. As discussed above, N‐doped carbon materials typically exhibit enhanced conductivity, large interlayer spacing, and additional K^+^ absorption sites. In this section, we highlight the recent progress in the synthesis and application of N‐doped carbon as anode materials for PIBs. Typical N‐doped carbon materials and their electrochemical performance as PIBs anode are summarized in **Table** **1**.

**Table 1:** Summary of nitrogen‐doped carbon as anode electrode reported recently and their corresponding electrochemical performance.

Materials	Electrolytes [solvents, v/v]	Capacity [mA h g^−1^]	Rate capacity [mA h g^−1^]	ICE [%]	Lifespan	Voltage window [V]	Refs.
N‐Doped graphitic nanocarbon	0.8 m KPF_6_ (Ethylene carbonate/EC: Diethyl carbonate/DEC = 1:1)	280 at 0.05 A g^−1^	56.6 at 5 A g^−1^	−	−	0.1–2.0	[57]
N‐Doped graphene (CVD)	0.8 m KPF_6_ (EC: DEC = 1:1)	270 at 0.1 A g^−1^	–	80	−96.5% after 50 cycles (0.1 A g^−1^)	0–1.5	[37]
N‐Doped graphene	0.8 m KPF_6_ (EC: DEC = 1:1)	320 at 50 A g^−1^	170 at 0.5 A g^−1^	37.29	−88% after 500 cycles (0.5 A g^−1^)	0–3.0	[100]
Honeycomb‐like N‐doped carbon	0.8 m KPF_6_ (EC: DEC = 1:1)	367.1 at 0.05 A g^−1^	91.8 at 10 A g^−1^	–	87.8% after 600 cycles (0.3 A g^−1^)‐	0.01–3.0	[81]
N‐Doped hierarchical porous carbon	3 m KFSI (Dimethyl ether/DME)	292 at 0.1 A g^−1^	94 at 10 A g^−1^	–	–	0.01‐3.0‐	[62]
Necklace‐like N‐doped hollow carbon	0.8 m KPF_6_ (EC: DEC = 1:1)	293.5 at 0.1 A g^−1^	204.8 at 2 A g^−1^	–	−74% after 1000 cycles (1 A g^−1^)	0.01–2.5	[101]
N‐Doped carbon nanosheets	0.8 m KPF_6_ (EC: DEC = 1:1)	440 at 0.3 A g^−1^	170 at 6 A g^−1^	–	−70% after 3000 cycles (5 A g^−1^)	0.01–2.0	[71]
Graphene‐like N‐doped carbon	0.8 m KPF_6_ (EC: DEC = 1:1)	312 at 0.2 C	133 at 18 C	46.21	–	0.01–3.0	[72]
N/O dual‐doped hard carbon	1 m KPF_6_ (Diglyme)	304.6 at 0.1 A g^−1^	178.9 at 5 A g^−1^	40.8	−74% after 5000 cycles (1 A g^−1^)	0.01–3.0	[102]
Amorphous hard carbon	3 m KFSI (Ethoxymethane)	294 at 0.05 A g^−1^	55 at 2 A g^−1^	51	−38% after 1000 cycles (1 A g^−1^)	0.005–3.0	[99]
N‐Doped carbon nanofibers	1 m KPF_6_ (EC: DEC = 1:1)	215.2 at 0.2 C	84.7 at 5 C	37.8	–	0.01–2.0	[103]
N‐Doped hard carbon microspheres	3 m KFSI (DME)	250 at 0.12 C	154 at 72 C	75	−75% after 4000 cycles (1.8C)	0.01–3.0	[104]
NOCNBs	0.8 m KPF_6_ (EC: DEC = 1:1)	468 at 0.05 A g^−1^	235 at 1.6 A g^−1^	49	92.8% after 1600 cycles (1 A g^−1^)	0.01–3.0	[105]
Hard–soft composite carbon	0.8 m KPF_6_ (EC: DEC = 1:1)	230 at 0.5 C	45 at 10 C	67	93% after 200 cycles (1C)	0.1–2.0	[106]
NC@CoP/NC	0.8 m KPF_6_ (EC: DEC = 1:1)	260 at 0.1 A g^−1^	200 at 2 A g^−1^	–	93% after 100 cycles (0.1 A g^−1^)	0.01–2.5	[107]
S/N‐Doped carbon	0.8 m KPF_6_ (EC: DEC = 1:1)	437 at 0.1 A g^−1^	72 at 10 A g^−1^	45	75% after 3000 cycles (2 A g^−1^)	0.01–3.0	[108]
Graphite	0.5 m KPF_6_ (EC: DEC = 1:1)	207 at 0.005 A g^−1^	141 at 0.2 A g^−1^	74.3	–	0.01–2.0	[47]
Graphitic carbon hollow nanocage	1 m KFSI (EC: PC = 1:1)	212 at 0.2 C	40 at 5 C	40	92% after 100 cycles (0.2 C)	0.01–3.0	[109]
N,P‐VG@CC	1 m KPF_6_ 5wt% FEC (EC: DEC = 1:1)	344.3 at 0.025 A g^−1^	156.1 at 2 A g^−1^	–	53 82% after 1000 cycles (0.025 A g^−1^)	0.01–3.0	[110]
Carbon dots@rGO	0.8 m KPF_6_ 5wt% Fluoroethylene carbonate/FEC (EC: DEC = 1:1)	310 at 0.1 A g^−1^	185 at 0.5 A g^−1^	44.4	90% after 840 cycles (0.2 A g^−1^)	0.01–3.0	[111]
Short‐Range order Mesoporous Carbon	0.8 m KPF_6_ (EC: DEC = 1:1)	286.4 at 0.05 A g^−1^	144.2 at 1 A g^−1^	63.6	70% after 1000 cycles (1 A g^−1^)	0.01–2.5	[71]
Hierarchical porous carbon	0.8 m KPF_6_ (EC: DEC = 1:1)	225 at 0.04 A g^−1^	79 at 0.8 A g^−1^	–	–	0.01–3.0	[112]
Amorphous hard carbon	1 m KPF_6_ (EC: PC = 1:1)	294 at 0.05 A g^−1^	55 at 2 A g^−1^	51	38% after 1000 cycles (1 A g^−1^)	0.005–3.0	[19]

### 3.1 Nitrogen‐Doped Graphitic Carbon Materials

**Figure 3:**
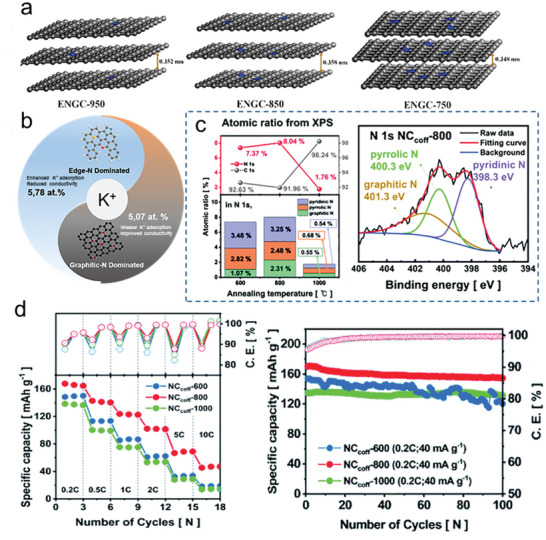
a) Schematic illustration of ENGC with different interlayer spacing prepared at different temperatures. Reproduced with permission.^[^ ^56^ ^]^ Copyright 2022, Elsevier. b) Schematic diagram comparing the effect of edge‐N and graphitic‐N in carbon anodes on K^+^ storage. c) Atomic ratio from the XPS for the prepared NC_coff_ samples (right) and N 1s XPS spectra of NC_coff‐800_ (left). d) Rate capability at various current densities from 0.04 to 2 A g^−1^ for the prepared NC_coff_ electrodes (right) and NC_coff‐600_, NC_coff‐800,_ and NC_coff‐1000_ at 40 mA g^−1^ (left). c,d) Reproduced with permission.^[^ ^58^ ^]^ Copyright 2022, Royal Society of Chemistry.

Inspired by the K^+^ storage capability of graphite, many graphitic carbon materials have been synthesized and studied as anode materials for PIBs. However, the interlayer spacing of these graphitic carbon materials is very similar to that of graphite. As a result, when they are used as PIBs anode, they are subject to the same limitations as graphite, i.e., huge volume expansion and poor K^+^ storage kinetics. Li et al. demonstrated that nitrogen doping can efficiently regulate the interlayer spacing of graphitic carbon materials.^[^ ^56^ ^]^ Through adjusting the carbonization temperature, a series of N‐doped graphitic carbon materials (denoted as ENGC‐T) were synthesized and it has been found that the doped nitrogen atoms were mainly in the form of pyrrolic‐N. As mentioned in Section 2, the *p* orbital lone pair in pyrrolic‐N is perpendicular to the graphene plane, which enables to enlarge the interlayer spacing. Therefore, all ENGC‐T had a larger interlayer spacing than that of graphite (0.335 nm). Moreover, it found that the content of pyrrolic‐N positively correlated with the variation of interlayer spacing. Finally, ENGC‐850 with an ultra‐high pyrrolic‐N content (42 at. %) had the largest interlayer spacing of 0.358 nm (**Figure** **3**a). Attributing to the large interlayer spacing, ENGC‐850 delivered a high capacity (319.6 mAh g^−1^ at 0.1 A g^−1^), an excellent rate capacity (228.9 mAh g^−1^ at 2 A g^−1^), and a prolonged cycle lifespan (188.9 mAh g^−1^ over 2200 cycles). In addition to enlarging the interlayer spacing, nitrogen doping can generate additional active sites for K^+^ storage. As known, pyridinic‐N and pyrrolic‐N usually located at the edges of graphitic carbon nanodomains to form dangling nitrogen bonds. K^+^ can be bonded to these dangling nitrogen bonds, thus enhancing charge storage capacity. Alshareef et al. synthesized a kind of N‐doped graphitic nanocarbon (GNC).^[^ ^57^ ^]^ By tuning the annealing temperature, the contents of edge‐nitrogen in GNC were controlled. When the annealing temperature was 600 °C, the as‐obtained GNC600 displays a high content of edge‐nitrogen reaching up to 72.2% (pyridinic‐N, 28.9 at. %; pyrrolic‐N, 43.3 at. %, respectively). Electrochemical characterizations indicated that the reversible capacity of GNC600 was higher than that of other designed graphitic carbon materials due to the high content of edge‐nitrogen. At a current density of 0.05 A g^−1^, the capacity was 280 mAh g^−1^, and when the current density was increased to 0.2 A g^−1^, the capacity remained 189 mAh g^−1^ after 200 cycles. Beyond capacity, the rate performance is another critical performance for anode materials. Graphite materials typically exhibit poor rate performance due to their limited electrical conductivity (3 × 10^3^ S cm^−1^). Introducing a high content of graphitic‐N can significantly enhance the electrical conductivity of graphitic carbon materials. Hwang et al. prepared an N‐doped carbon matrix (NC_coff_) by calcination of coffee grounds.^[^ ^58^ ^]^ NC_coff‐600_, NC_coff‐800_, and NC_coff‐1000_ were obtained at calcination temperatures of 600, 800, and 1000 °C, respectively. The content of nitrogen in NC_coff‐800_ was the highest (Figure 3c, 8.04 at. %). Relative to other samples, the best conductivity (6.96 × 10^4^ S cm^−1^) comes from the high graphitic‐N content in NC_coff‐800_, and thus the rate performance of NC_coff‐800_ was superior to that of NC_coff‐600_ and _NCcoff‐1000_ (Figure 3d right). Due to the high nitrogen content, the NC_coff‐800_ exhibited superior initial capacity (170.29 mAh g^−1^) and stable cycling (Figure 3d left) with a 90.4% capacity retention over 100 cycles. Additionally, ensuring a high Coulombic efficiency is essential for anode materials, as it helps prevent irreversible potassium deposition. Since graphitic‐N can cause irreversible potassiation reactions, the edge‐nitrogen content should be increased to enhance the initial Coulombic efficiency (ICE) of N‐doped graphitic carbon.^[^ ^35^ ^]^ Alshareef et al. demonstrated a site‐selective doping strategy to control edge‐nitrogen doping in carbonaceous materials.^[^ ^59^ ^]^ When the pyrolysis temperature was 500 °C, the as‐prepared ENDC500 had a high nitrogen‐doping level (up to 10.5 at. %) and a high edge‐nitrogen ratio (87.6 %: 32.7%pyridinic‐N and 54.9% pyrrolic‐N). The high nitrogen doping level can be achieved by the sequential decomposition of radicals from pyrrole and aniline in polyaniline‐co‐polypyrrole (PACP). As a result, the ENDC500 electrode displays a high initial Coulombic efficiency of 65% (the first cycle discharge/charge capacity is 828/481 mAh g^−1^). The ENDC500 anode shows a reversible capacity of 305 mAh g^−1^ after 660 cycles at 0.2 A g^−1^ (93.8 % capacity retention). Similarly, Fahlman et al. reduced the content of graphitic‐N in nitrogen‐deficient g‐C_3_N_4_ (ND‐g‐C_3_N_4_) by magnesiothermic denitriding technology.^[^ ^30^ ^]^ The graphitic‐N content in ND‐g‐C_3_N_4_ decreased from 23.03 at. % to 17.8 at. % through magnesiothermic denitriding. The ND‐g‐C_3_N_4_ with less graphitic‐N (17.8 at. %) has a promising initial Coulombic efficiency. Subsequently, the ICE of ND‐g‐C_3_N_4_ electrode has increased to 82.74%. The difference between edge‐nitrogen and graphitic‐N should be clearly seen. Controlling the amount of doped nitrogen in different positions can distinguish the function of various nitrogen‐doped types. Sun and co‐workers reported throughout the precise preparation of high‐content edge‐N doped carbon (HENC) and high‐content graphitic‐N doped carbon (HGNC) harnessing basically identical N‐doping levels (Figure 3b, 5.78 at. % for HENC; 5.07 at. % for HGNC).^[^ ^60^ ^]^ This design enables us to distinctly separate the function of different types of N species and precisely identify the specific effects of each type. The concept of K^+^ adsorption energy (Ea) has been widely adopted to illustrate the distinctions among different types of doped nitrogen clearly. According to the result of first‐principles calculations, the E_a_ of pristine graphene, pyridinic‐N, pyrrolic‐N, and graphitic‐N is −0.26, −2.26, −2.09, and −0.01 eV, respectively. With respect to the bond length, the nearest distance between K and N‐C is 2.62, 2.73, 3.07, and 3.03 Å on the pyridinic‐N, pyrrolic‐N, graphitic‐N and pristine graphene, respectively. High adsorption energy suggests that the above material can effectively trap and store K^+^. A shorter distance indicates a stronger interaction between K and graphene. As a result, the edge‐nitrogen will enhance K^+^ adsorption, while the graphitic‐N can weaker K^+^ adsorption. In summary, pyridinic‐N and pyrrolic‐N are edge‐nitrogen. Boosting the ratio of edge‐nitrogen doping enhances the initial Coulombic efficiency of N‐doped graphitic carbon. Individually, the presence of pyrrolic‐N facilitates an expansion of the interlayer spacing. Pyridinic‐N in graphitic carbon materials increases the number of active sites. The content of graphitic‐N should be optimized, on the one hand, graphitic‐N can lead to low ICE and poor cycling stability. On the other hand, the conductivity can increase by improving the graphitic‐N content.

**Figure 4:**
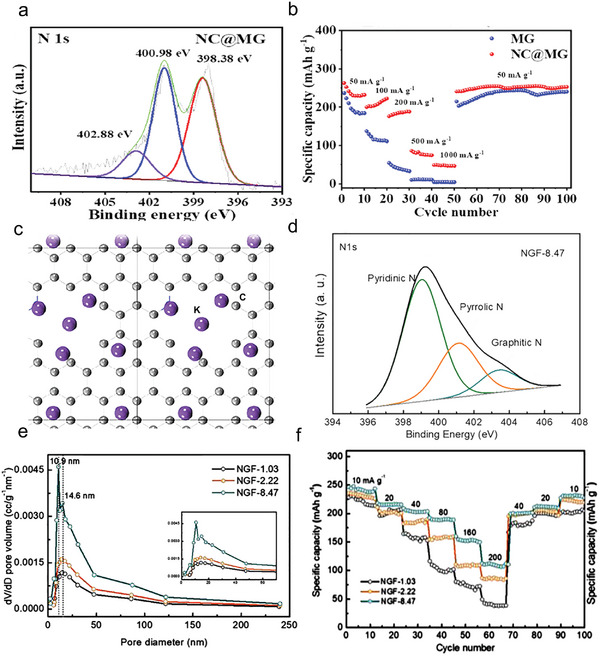
a) The high‐resolution XPS N 1s spectra of NC@MG. b) Rate performance of MG and NC@MG at different current densities. a,b) Reproduced with permission.^[^ ^67^ ^]^ Copyright 2021, Elsevier. c) Theoretical analysis of K^+^ adsorption and specific capacity of hole carbon structure with the N‐doping. d) The high‐resolution XPS N 1s spectra of NGF‐8.47. e) The pore size distribution of NGF‐1.03, NGF‐2.22 and NGF‐8.47. f) Rate performance evaluations of NGF‐1.03, NGF‐2.22 and NGF‐8.47. c–f) Reproduced with permission.^[^ ^68^ ^]^ Copyright 2019, Elsevier.

In recent years, graphene has garnered significant research interest due to its unique monolayer structure and exceptional physical properties.^[^ ^48^, ^61^, ^62^, ^63^ ^]^ Graphene is limited for further applications as PIB anode because of its low capacity. First‐principle calculations reveal that among the three types of doped nitrogen, pyridinic‐N exhibits the strongest affinity for K^+^, while graphitic‐N shows the weakest affinity.^[^ ^64^, ^65^, ^66^ ^]^ The strongest affinity for K^+^ can lead to high capacity of graphene. Consequently, high concentration of pyridinic‐N increases the capacity of graphene. Pint et al. investigated high amount of pyridinic‐N (42.4 at. %) in few‐layer N‐doped graphene for PIBs displays an exceptionally high affinity for K^+^ due to the high K^+^ adsorption energy.^[^ ^37^ ^]^ The N‐doped graphene achieves a high capacity due to the high content of pyridinic‐N. The N‐doped graphene has reported that the K^+^ storage capacity is as high as 350 mAh g^−1^ at 0.05 A g^−1^, which is extremely over to the graphite anode's theoretical capacity (278 mAh g^−1^) in PIBs. Additional experiments on the cycling performance at 0.1 A g^−1^ indicate a stable and high capacity, which began at 270 mAh g^−1^ and 57% capacity retention after 100 cycles. Similarly, Nan et al. designed and synthesized the N‐doped carbon nanosheets coated multilayer graphite (NC@MG) anode materials.^[^ ^67^ ^]^ Benefiting from the coating of highly pyridinic‐N content (45.74 at. %, **Figure** **4**a), the capacity and rate capacity of NC@MG are much better than MG (Figure 4b). The multilayer graphite (MG) and NC@MG exhibit specific charge capacity of 215.7 and 252.7 mAh g^−1^, respectively, after 200 cycles at 0.05 A g^−1^. Significantly, the NC@MG demonstrates a high reversible capacity of 221.2 mAh g^−1^ at 0.2 A g^−1^ for PIBs. As the nitrogen doping content increases, the percentage of pyridinic‐N and pyrrolic‐N also rises, leading to the formation of holey structures with more active sites that enhance rate capacity. Shen et al. reported large pore volume can enhance the rate capacity of the graphene carbon material.^[^ ^68^ ^]^ As shown in Figure 4c, with the increase of K^+^ concentration, K^+^ could be trapped nearby the hole first and then adsorbed on hollow sites of carbon plane. According to the report, the holey structures can directly improve the capacity. For instance, in N‐doped graphitic foam (NGF) materials, different nitrogen‐doping carbon materials were synthesized through controlling the annealing time. NGF‐1.03, NGF‐2.22, and NGF‐8.47 were produced by maintaining the furnace at 1000 °C for 30, 10, and 20 min, respectively. The NGF‐8.47 occurred a highly doped nitrogen level of 8.47 at. % when the annealing time is 20 min at 1000 °C (Figure 4d). Benefit from the high amount of edge‐nitrogen (89 at. %), the pore volume of NGF‐8.47 (0.212 cm^3^ g^−1^) is largest than NGF‐1.03 (0.085 cm^3^ g^−1^) and NGF‐2.22 (0.118 cm^3^ g^−1^). Specifically, NGF‐8.47 contains large mesopores, i.e., 10.9 and 14.6 nm pores, compared to NGF‐1.03 and NGF‐2.22 (Figure 4e). It is easy to accommodate more K^+^ and facilitate contact between electrolyte and electrode through the increased pore volume originates from large amounts of mesopores. The NGF‐8.47 shows a good rate performance (Figure 4f). The specific capacity of low N‐doping samples (NGF‐1.03, NGF‐2.22) decays rapidly from 226 and 213 to 36 and 87 mAh g^−1^, respectively, whereas it remains relatively high capacity (112 mAh g^−1^ for NGF‐8.47), with increasing the charge/discharge rate from 10 to 200 mA g^−1^. Equally, graphene also has the property of a hydrophobic interface that leads to poor surface wettability between the electrode and the electrolyte, and nitrogen doping can improve surface wettability through permanent dipoles generate from C─N bonding.^[^ ^69^ ^]^ The permanent dipoles, which arise primarily from the electronegativity differences between nitrogen and carbon atoms in nitrogen‐doped carbon materials, leading to uneven electron distribution. The uneven electrode distribution can form stronger interactions with polar liquid molecules, enhancing the materials’ surface wettability. Lu et al. reported nitrogen doping can adjust the surface wettability of graphene tubes.^[^ ^70^ ^]^ The contact angle measurements revealed that N‐doped graphene tubes have hydrophilic surfaces with an initial contact angle of 64.1°, allowing rapidly diminishing contact angle. In contrast, the undoped graphene tubes exhibit hydrophobic characteristics with a contact angle of 128.9°, which remains a stable contact angle. Thus, the surface wettability can be optimized by nitrogen doping of graphene materials. In summary, first, in N‐doped graphene, the pyridinic‐N is most suitable for K^+^ storage, while the graphitic‐N is weakest for K^+^ affinity. Then, the increased pore volume can enhance the capacity by high levels of pyridinic‐N. Lastly, the surface wettability can be optimized by permanent dipoles in N‐doped graphene materials.

### 3.2 Nitrogen‐Doped Hard Carbon Materials

**Figure 5:**
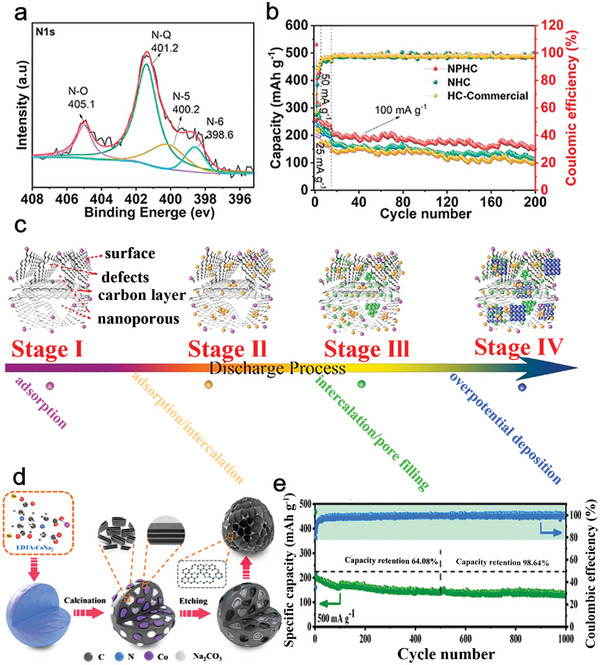
a)The NPHC XPS spectra of N 1s. b) Cycling properties at the current density of 25, 50, and 100 mA g^−1^. c) Schematic diagram of four different stages of the discharge process. a–c) Reproduced with permission.^[^ ^74^ ^]^ Copyright 2021, Elsevier. d) Scheme for the synthesis route of NGHCs from EDTA disodium cobalt salt hydrate. e) Long‐term cycle properties of NGHC‐750 at high rate of 0.5 A g^−1^. d,e) Reproduced with permission.^[^ ^75^ ^]^ Copyright 2020, Elsevier.

The structure of hard carbon consists of randomly dispersed graphitized microdomains. Graphitization is very difficult in hard carbon due to the sufficiently cross‐linked structure, which prefers to preserve the amorphous structure and inhibits the formation of the graphitic structure.^[^ ^26^, ^27^, ^33^ ^]^ In contrast to graphite, where K^+^ is compressed into restricted interlayer spacing, hard carbon typically has large interlayer spacing over short distances.^[^ ^25^, ^71^, ^72^, ^73^ ^]^ However, the short‐range ordered structures are discontinuous structures, which can block the conductive network and slow the speed of electronic transmission. N‐doped hard carbon material is one of promising anode materials to solve this challenge. Nitrogen doping not only enhances the capacity of K^+^ storage but also enhances the electrical conductivity of the hard carbon materials. On the one hand, as shown in **Figure** **5**a,b, Zhao et al. demonstrated the introduction of heteroatoms into hard carbon (HC) can enhance the storage mechanism of hard carbon materials to improve the capacity.^[^ ^74^ ^]^ The nitrogen‐doped hard carbon (NHC) with 3D hierarchical porous frameworks was synthesized through composite calcination technique (1300 °C). The amount of doped nitrogen in NHC is 0.8 at. %. The NHC demonstrates remarkable stability in PIBs, with only slight reductions in capacity seen after five and ten cycles at 0.025 and 0.05 A g^−1^, respectively. Furthermore, from the 21st to the 200th cycle at 0.1 A g^−1^, the capacity of NHC, and commercialized HC remains at 69.4%, and 65.2%, respectively. A model of the HC storage K^+^ mechanism is shown in Figure 5c. The mechanism can be simple division with four stages: adsorption, adsorption/intercalation, intercalation/pore filling, overpotential deposition. Each stage corresponding to a certain capacity. Nitrogen doping can increase the second‐stage capacity due to the form of defects, which provide reaction sites for K^+^. Similarly, high content of pyridinic‐N and pyrrolic‐N can from more defects in N‐doped hard carbon. Thus, the high amount of pyridinic‐N and pyrrolic‐N can significantly enhance the capacity. Zhang et al. synthesized the N‐doped and partly graphitized hard carbons (NGHCs) through ethylenediaminetetraacetic acid (EDTA) disodium cobalt salt hydrate (Figure 5d).^[^ ^75^ ^]^ Controllably adding graphitic domains and nitrogen elements to NGHCs is possible via a simple annealing process. The doped nitrogen content of NGHC‐650, NGHC‐750, and NGHC‐850 were 2.0, 0.78, 0.48 at. %, respectively. However, high graphitic‐N content (42.6%) in NGHC‐650 leads to poor cycle stability. Compared to graphitic‐N, high content of pyridinic‐N (41%) and pyrrolic‐N (24.6%) in NGHC‐750 introduce more defects and exhibit greater chemical reactivity, thereby enhancing the capacity for PIBs. The NGHC‐750 has a high reversible capacity of 298.8 mAh g^−1^ at 0.05 A g^−1^ and a steady cycle of 137.6 mAh g^−1^ at 0.5 A g^−1^ after 1000 cycles (Figure 5e). On the other hand, Agrawal et al. reported that nitrogen doping can optimize the electrical conductivity of hard carbon materials.^[^ ^76^ ^]^ By comparing the electrical conductivity of N‐doped nano‐carbon spheres (NNCS, 204 S m^−1^) with that of nano‐carbon spheres (NCS, 124 S m^−1^), it is demonstrated that nitrogen doping can enhance the electrical conductivity of nano‐carbon spheres. The content of doped nitrogen is 9.12 at. %. After nitrogen doping, nano‐sized carbon spheres show an increase of 39.5% in capacity. Among these two electrodes, the NNCS shows high discharge capacity of 286 mAh g^−1^. The specific capacity of 244 mAh g^−1^ was retained 85% over 200 cycles at 0.03 A g^−1^.

**Figure 6:**
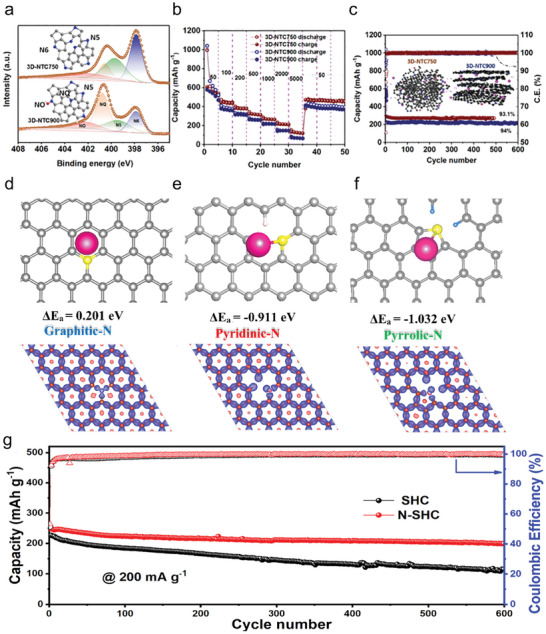
a) N 1s high‐resolution XPS spectra of 3D‐NTC. b) Rate performance of 3D‐NTC. c) Long‐term GCD cycling stabilities of 3D‐NTC at a current density of 1.0 A g^−1^. The DFT illustrations of adsorption energy of K^+^ (set adsorption energy at original hard carbon as 0 eV) and differential charge densities in (d) graphitic‐N, (e) pyrindic‐N, and (f) pyrrolic‐N defects. (g) Long‐term cycle properties at 0.2 A g^−1^ for N‐SHC and SHC electrodes. a–c) Reproduced with permission.^[^ ^42^ ^]^ Copyright 2020, Wiley‐VCH. d–g) Reproduced with permission.^[^ ^73^ ^]^ Copyright 2022, Multidisciplinary Digital Publishing Institute.

It is the same with graphitic carbon, the edge nitrogen also can improve the cycle capacity in N‐doped hard carbon. Alshareef et al. reported prepare carbon anodes with ultrahigh edge‐nitrogen doping through a new synthesis strategy for high‐performance PIBs.^[^ ^42^ ^]^ The obtained 3D N‐doped turbostratic carbon (3D‐NTC) possesses a 3D framework composed of carbon nanosheets, turbostratic crystalline structure, and ultrahigh edge‐doped nitrogen level. The amount of edge‐doped nitrogen was up to 16.8 at. % (**Figure** **6**a, 73.7% of total 22.8 at. % doped nitrogen). Benefiting from the high edge‐nitrogen doping (pyridinic‐N, 41.44%, and pyrrolic‐N, 32.41%) ratio of 3D‐NTC750, the 3D‐NTC750 anode displays a high capacity of 473 mAh g^−1^, and a high capacity retention of 93.1% over 500 cycles (Figure 6b,c). Thus, ultrahigh edge N‐doped hard carbon materials exhibit exceptional cycling capacity. In other research, Ci et al. have illustrated the edge‐nitrogen doping, as chemical active sites, are likely to act as additional defects with a higher affinity for attracting and storing more K^+^.^[^ ^52^ ^]^ A straight forward technique for producing N‐doped hierarchically porous carbon (NPC) was utilized by combining sodium citrate and urea. The relative content of pyridinic‐N, pyrrolic‐N, and graphitic‐N are 20.6, 40.9, and 38.5%, respectively. The NPC exhibited superior performance by high amount of edge‐doped nitrogen (61.5%). As anode materials, the as‐obtained NPC electrode demonstrated exceptional electrochemical performance, including ultrastable cyclability (384.2 mAh g^−1^ at 0.1 A g^−1^ after 500 cycles) and high rate capability (185 mAh g^−1^ at 10 A g^−1^). The capacity surpassed that of previously reported carbonaceous electrodes. Similarly, Tang et al. prepared an N‐doped carbon nanosheets anode material (UNCN) with ultrahigh nitrogen content (22.7 at. %).^[^ ^77^ ^]^ The amount of pyridinic‐N, pyrrolic‐N, and graphitic‐N are 42.1, 34.3, and 23.6%, respectively. The content of edge‐nitrogen doping up to 76.4%, which can contribute to the store of K^+^ through additional active sites. As a result, the UNCN electrode with high content of edge‐nitrogen doping exhibited a high capacity of 410 mAh g^−1^ at 0.5 A g^−1^. The battery exhibits capacity retention of 70% after 3000 cycles at 5.0 A g^−1^. The affinity of K^+^ is another important factor affecting the storage capacity of K^+^, which can be compared by the K^+^ adsorption energies (E_a_). According to the K^+^ adsorption energies (E_a_), the graphitic‐N can weaken K^+^ adsorption while the pyridinic‐N can have stronger K^+^ adsorption in N‐doped hard carbon. Xie et al. reported that the K^+^ adsorption energies of pristine carbon, graphitic‐N doped carbon, and pyridinic‐N doped carbon were −0.229, −0.024, and −0.284 eV, respectively, by simulations.^[^ ^78^ ^]^ The DFT calculations indicated that pyridinic‐N enhances the ability of K^+^ adsorption. Therefore, K^+^ absorbed at the pyridinic‐N sites would be easier to absorb and with high abundance. The content of pyridinic‐N in the N‐doped carbon microsphere (NCS) is 76%, which is high in N‐doped carbon microsphere. Therefore, the reversible capacity (170 mAh g^−1^) was exhibited when cycling around at 0.5 A g^−1^. Yang et al. evaluated the K^+^ adsorption energy and charge densities for three kinds of nitrogen doping surface defects on hard carbon by DFT calculations (Figure 6d–f).^[^ ^73^ ^]^ The DFT calculations revealed distinct K^+^ adsorption energies for different nitrogen‐doping types in graphitic carbon. Graphitic‐N showed a low adsorption energy of 0.201 eV, indicating weaker K^+^ attraction, while pyridinic‐N and pyrrolic‐N exhibited significantly higher adsorption energies of – 0.911 and −1.032 eV, respectively. This suggests that pyridinic‐N and pyrrolic‐N have a stronger tendency to attract and retain K^+^. The introduction of N‐doping enhanced the ability of K^+^ adsorption at these sites. This can positively contribute to the capacitive performance and reversible capacity of the N‐doped hard carbon (N‐SHC) samples. The charge density analysis reveals distinct electron distribution patterns around various doped niteogen sites in N‐doped hard carbon. Figure 6d,e (bottom image) reveal that pyridinic‐N and pyrrolic‐N defects exhibit heterogeneous electron distributions and electron vacancies, which promote stronger interactions with K^+^. Conversely, the electron‐rich environment around the graphitic‐N site leads to a lower tendency for K^+^ adsorption, which corresponds to its lower adsorption energy (Figure 6f). Above all, the nitrogen‐doped hard carbon anode material has exhibited high cycle stability. The cycling stability of N‐SHC is better than that of undoped SHC. After 100 cycles at 0.2 A g^−1^, the capacity of N‐SHC reaches 224 mAh g^−1^ with 90% capacity retention. The reversible capacity of SHC rapidly decays to 178 mAh g^−1^ with 78% capacity retention at the same current density. Based on the understanding of K^+^ affinity, it is crucial to consider that pore structure significantly influences the diffusion and adsorption behavior of K^+^. Yu et al. fabricated an interconnected N‐doped hierarchical porous carbon nanostructure.^[^ ^79^ ^]^ They have evaluated the diffusion ability of potassium on macroporous and microporous structures through the K^+^ adsorption energy. The adsorption energies of potassium on the most stable sites of macroporous pyridinic‐N, pyrrolic‐N, and graphitic‐N structures are −1.17, −1.37, and −2.15 eV, respectively. In order to evaluate K^+^ diffusion on macroporous and microporous structures, the differences in adsorption energy between the most stable sites and their corresponding alternative sites are calculated. The adsorption energy difference (ΔEa) results are 0.41, 0.34, and 1.31 eV for pyridinic‐N, pyrrolic‐N, and graphitic‐N structures. These results indicate that the diffusion of K^+^ in macroporous N‐HPC is faster than that in microporous structures. Additionally, the adsorption energy of potassium on N‐doped porous carbon is more favorable than that on undoped carbon (−0.60 eV). Thus, the macroporous N‐HPC anode materials showed a superior reversible capacity of 292 mAh g^−1^ at 0.1 A g^−1^, excellent rate capability (94 mAh g^−1^ at 10.0 A g^−1^), and outstanding cycling stability (157 mAh g^−1^ after 12 000 cycles at 2.0 A g^−1^).

In summary, in hard carbon materials, nitrogen doping not only enhances the capacity of K^+^ storage but also enhances the electrical conductivity of the hard carbon materials. Pyridinic‐N and pyrrolic‐N can be referred to as edge‐doped nitrogen, contributing to abundant defects. The hard carbon with ultrahigh edge nitrogen‐doped carbon materials exhibited excellent cycling capacity. Pyridinic‐N and pyrrolic‐N in N‐doped macroporous hard carbon materials can easily be used for K^+^ diffusion. Meanwhile, graphitic‐N can weaken K^+^ adsorption, while pyridinic‐N can have stronger K^+^ adsorption in hard carbon.

### 3.3 Nitrogen‐Doped Soft Carbon Materials

**Figure 7:**
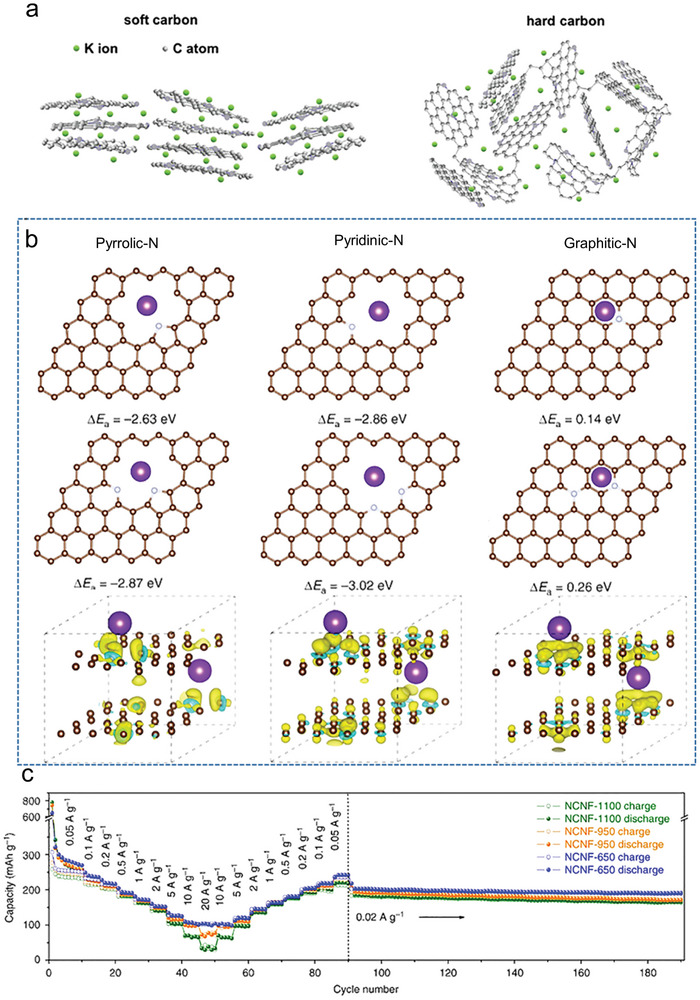
a) Schematic illustration of the difference between N‐doped soft carbon and N‐doped soft. a) Reproduced with permission.^[^ ^80^ ^]^ Copyright 2020, Elsevier. b) Theoretical simulations of K‐adsorption in different N‐doped structures. Yellow and blue areas represent increased and decreased electron density, respectively. Brown, silver, and purple balls represent carbon, nitrogen, and potassium atoms, respectively. c) Rate performance of NCNFs with rates ranging from 0.05 to 20 A g^−1^. c,d) Reproduced with permission.^[^ ^39^ ^]^ Copyright 2018, Nature Publishing Group.

In **Figure** **7**a, the differences between soft carbon and hard carbon materials are illustrated. Hard carbon exhibits a highly disordered amorphous structural characteristic. In contrast, soft carbon possesses a long‐range ordered structure similar to graphite, which can convert to graphite by heat treatment ≈2300 °C. Soft carbon materials are particular interest due to their long‐range ordered structure.^[^ ^19^, ^80^ ^]^ The limited rate performance of soft carbon materials significantly hinders their development and application in high‐performance active materials. The K^+^ adsorption energy and the interlayer spacing together boost the rate performance of soft carbon anode materials. As an early explorer of N‐doped soft carbon materials, Lei et al. reported highly N‐doped carbon nanofibers (NCNF) with superior rate capability and cyclability for use as anode materials in PIBs. Different soft carbon materials (NCNF‐650, NCNF‐950, NCNF‐1100) were obtained according to different carbonization temperatures (650, 950, 1100 °C). The highest content of nitrogen doping (13.8 at. %) appeared in samples of NCNF‐650. The surface defects can easily form once the pyridinic‐N and pyrrolic‐N was formed in soft carbon. Meanwhile, the surface defects of soft carbon typically serve as alkali‐ion storage sites, thereby high pyrrolic‐N content can facilitate rapid kinetics and enhancing rate performance. According to the K^+^ adsorption energy, the potassium adsorption energy of pyridinic‐N (−3.02 eV) and pyrrolic‐N (−2.87 eV) is significantly higher than that of graphitic‐N (0.26 eV). Above all, due to the highest K^+^ adsorption energy (E_a_ = −3.02 eV, Figure 7b), the largest graphene interlayer (pyrrolic‐N content, 22.7 at. %), and the resultant carbon defects (nitrogen element content, 13.8 at. %), the NCNF‐650 anode delivers reversible capacities of 368 mAh g^−1^ at 0.025A g^−1^ and 101 mAh g^−1^ at 20 A g^−1^, and retains 146 mAh g^−1^ at 2 A g^−1^ after 4000 cycles (Figure 7c).^[^ ^39^ ^]^ In the nitrogen doping soft carbon, high pyrrolic‐N content can also enlarge interlayer spacing. Qiu et al. synthesized N ‐doped soft carbon nanocapsules (NSCNs) by the MgO template method. The relative content of pyridinic‐N, pyrrolic‐N, and graphitic‐N in NSCNs is 32.8, 42.5, and 24.7%, respectively.^[^ ^80^ ^]^ Pyrrolic‐N changes the interlayer spacing by electrostatic repulsion between layers. Due to the high content of pyrrolic‐N, the interlayer spacing of NSCNs attached to 0.356 nm. Meanwhile, the NSCNs electrode allowed for the rapid diffusion and transfer of K^+^ and electrons through the defects in the formation of pyridinic‐N and pyrrolic‐N. As a result, the NSCNs delivered a large capacity of 293 mAh g^−1^ at 0.05 A g^−1^ and retained ≈85.5% of the initial capacity after 500 cycles at 1.0 A g^−1^. The NSCNs delivers reversible charge capacities of 293, 266, 246, 216, 194, 174, and 151 mAh g^−1^ at 0.05, 0.1, 0.2, 0.5, 1, 2, and 5 A g^−1^, respectively. Similarly, Li et al. prepared honeycomb‐like N‐doped carbon (N‐C) nanosheets by growing MOFs on LDH nanosheets followed by pyrolysis acid‐etching steps.^[^ ^81^ ^]^ The contents of pyrrolic‐N in N‐C is 35.9 at. %. Similarly, the highest pyrrolic‐N content of ZIF‐67‐derived N‐C material with the relative proportions is 34.1%. The corresponding interlayers spacing is 0.376 nm, which is larger than the ZIF‐67‐derived N‐C material (0.269 nm). After 2000 cycles, the anode fabricated from N‐C nanosheets exhibited outstanding cycling performance with a reversible discharge capacity of 143 mAh g^−1^ at 1.0 A g^−1^ and acceptable rate capability (91.8 mAh g^−1^) up to 10 A g^−1^. The outstanding performance is mainly ascribed to high amount of pyridinic‐N and pyrrolic‐N, which enhance the conductivity and enlarges the interlayer spacing. In summary, the adsorption energy of pyridinic‐N and pyrrolic‐N for K^+^ is much higher than that of graphitic‐N. The abundance of pyridinic‐N can significantly increase the electrical conductivity of soft carbon materials. The content of pyrrolic‐N can adjust the interlayer spacing of soft carbon materials.

## 4 Nitrogen‐Doped Carbon Modification as Anode Materials

In other respects, N‐doped carbon materials are often used as functional layer materials to modify and optimize other anode materials (metal oxide/selenides/sulfides, potassium metal etc.) that are not directly used as anode materials. When N‐doped carbon materials as functional layer modification of anode materials, the N‐doped carbon materials have two main functions. First, N‐doped carbon can improve the material's conductivity by the conductive network of N‐doped carbon materials. Second, N‐doped carbon can buffer the volume expansion of anode materials through the porous structure. The section will present the recent progress in N‐doped carbon as modification materials to optimize the electrochemical performance of non‐carbonaceous anode material. Meanwhile, the non‐carbonaceous anode materials modified with N‐doped carbon details in compositions, type of reaction, and electrochemical properties are summarized in **Table** **2**.

**Table 2:** Summary of nitrogen‐doped carbon as functional layer modification anode materials reported recently and their corresponding electrochemical performance.

Materials	Electrolytes [solvents, v/v]	capacity (mA h g ^−1^)	Rate Capacity [mA h g^−1^]	ICE [%]	Lifespan	Voltage window [V]	Refs.
N‐MoSe_2_@carbon	0.8 m KPF_6_ (EC: DEC = 1:1)	433 at 0.1 A g^−1^	169 at 5 A g^−1^	–	84.2% after 5000 cycles (5 A g^−1^)	0.01–3.0	[113]
FeS_2_@NC nanosheets	1.0 m NaPF_6_ (DME)	812 at 0.1 A g^−1^	400 at 5 A g^−1^	54.3	−77.2% after 350 cycles (1.0 A g^−1^)	0.01–3.0	[114]
Cu_2_S@NC	5 m KFSI (DME)	317 at 1 A g^−1^	257 at 6 A g^−1^	57.4	83.2% after 800 cycles (0.5 A g^−1^)	0.01–2.6	[115]
NiCo_2_S_4_@N‐HCNF	2.0 m KOH	349.4 at 0.1 A g^−1^	197.9 at 3.2 A g^−1^	53.3	−64% after 600 cycles (3.2 A g^−1^)	0.01–3.0	[116]
Bi@N‐CT	1.0 m KFSI (EC:DEC = 1:1)	316 at 1 C	297 at 20 C	–	−76.88% after 1000 cycles (10 C)	0.01–1.5	[117]
Red P@N‐PHCNFs	0.7 m KPF6 (EC:DEC = 1:1)	745 at 0.1 A g^−1^	565 at 5 A g^−1^	–	52% after 800 cycles (5 A g^−1^)	0.01–2.0	[118]
MnMoO_4_@NC	0.8 m KPF_6_ (EC: DEC = 1:1)	220.4 at 0.5 A g^−1^	83 at 1 A g^−1^	32.4	43% after 400 cycles (0.5 A g^−1^)	0.01–3.0	[119]
Yolk–shell FeS/MoS_2_@N‐doped carbon nanocubes	0.8 m KPF_6_ (EC: DEC = 1:1)	342 at 0.1 A g^−1^	295 at 0.5 A g^−1^	–	68% after 10 000 cycles (1.0 A g^−1^)	0.01–2.5	[45]
CuO/Cu‐NCNFs	0.8 m KPF_6_ (EC: DEC = 1:1)	417 at 0.1 A g^−1^	87.5 at 2 A g^−1^	55.3	49% after 100 cycles (0.1 A g^−1^)	0.01–3.0	[120]
Co_3_O_4_@N‐Doped Carbon	0.5 m KPF_6_ (EC: DEC = 1:1)	1229.2 at 0.05 A g^−1^	408.2 at 2 C	48.2	34% after 740 cycles (0.5 A g^−1^)	0.01–3.0	[85]
MoSe_2_/N−C	0.8 m KPF_6_ (EC: DEC = 1:1)	222.8 at 0.1 A g^−1^	94.2 at 10 A g^−1^	41	77% after 400 cycles (0.5 A g^−1^)	0.01–2.5	[121]
Fe_2_VO_4_⊂NC nanopeapods	0.8 m KPF_6_ (EC: DEC = 1:1)	394 at 0.1 A g^−1^	159 at 4 A g^−1^	60.8	89% after 2300 cycles (4.0 A g^−1^)	0.01–2.6	[84]
MoS_2_/NDG	0.75 m KPF_6_ (EC: DEC = 1:1)	486 at 0.1 A g^−1^	176.6 at 2 A g^−1^	59.3	−45% after 150 cycles (1.0 A g^−^1)	0.01–3.0	[31]
3D SnSb@NC	0.8 m KPF_6_ (EC: DEC = 1:1)	357.2 at 0.05 A g^−1^	116.6 at 2 A g^−1^	–	−24% after 200 cycles (0.5 A g^−1^)	0.01–2.2	[87]
Multicore–shell structured Bi@N‐doped carbon	1.0 m KPF_6_ (Tetraethylene glycol dimethyl ether/TEGDME)	327 at 1.0 A g^−1^	152 at 100 A g^−1^	43	62% after 1000 cycles (10 A g^−1^)	0.1–1.5	[86]
E‐MoS_2_/NOC TC	0.8 m KPF_6_ (EC: DEC = 1:1)	397 at 0.25 A g^−1^	247 at 1 A g^−1^	44	44% after 500 cycles (10 A g^−1^)	0.1–3.0	[122]
Double‐shelled Ni‐Fe‐P/N‐doped carbon nanoboxes	3.0 m KFSI (TEGDME)	229.7 at 0.1 A g^−1^	75.6 at 1 A g^−1^	32.2	50% after 2600 cycles (10 A g^−1^)	0.01–3.0	[123]
Sb@CSN	0.8 m KPF_6_ (EC:DEC = 1:1)	551 at 0.1 A g^−1^	530 at 0.2 A g^−1^	61	94% after 90 cycles (0.1 A g^−1^)	0.01–2.4	[124]
Sb/CNS	0.8 m KPF_6_ (EC:DEC = 1:1)	395.9 at 0.05 A g^−1^	101.4 at 2 A g^−1^	48	90% after 600 cycles (0.2 A g^−1^)	0.01–2.5	[46]
MOF–NCNT	0.8 m KPF_6_ (EC:DEC = 1:1)	254.7 at 0.05 A g^−1^	102 at 2 A g^−1^	24.45	77.86% after 500 cycles (2 A g^−1^)	0.01–3.0	[125]
MOF–NPC	0.7 m KPF_6_ (EC:DEC = 1:1)	587.6 at 0.05 A g^−1^	186.2 at 2 A g^−1^	–	79.3% after 600 cycles (0.2 A g^−1^)	0.1–3.0	[126]

### 4.1 Nitrogen‐Doped Carbon Optimize the Conductivity of Anode Materials

**Figure 8:**
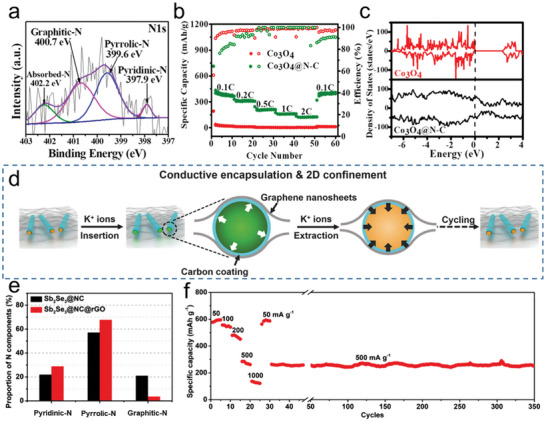
a) XPS spectra of N 1s of Co_3_O_4_@N‐C. b) The rate ate capability from 0.1 to 2 C of Co_3_O_4_@N‐C. c) DOS of Co_3_O_4_ and Co_3_O_4_@N‐C composite. a–c) Reproduced with permission.^[^ ^62^ ^]^ Copyright 2022, Elsevier. d) Schematics of the electrochemical process by combining encapsulation and confinement. e) Proportion of nitrogen components in pyridinic‐N, pyrrolic‐N, and graphitic‐N calculated by N 1s XPS spectra of Sb_2_Se_3_@NC and Sb_2_Se_3_@NC@rGO. f) Rate and long‐term cycling performance of the Sb_2_Se_3_@NC@rGO electrode. d–f) Reproduced with permission.^[^ ^70^ ^]^ Copyright 2018, Nature Publishing Group.

In anode materials, the conversion type of anode materials has emerged as a key focus of research due to their high capacity and low cost. In the conversion type of anode materials, poor electrical conductivity is the main challenge in many conversion anode materials (metal oxide/selenides/sulfides).^[^ ^31^, ^49^, ^82^, ^83^ ^]^ N‐doped carbon materials, when used as a functional layer, exhibit high electrical conductivity and can be integrated with conversion anode materials in PIBs to form composite materials.^[^ ^84^, ^85^ ^]^ The N‐doped carbon composite materials can help to improve the conductivity of the conversion anode material. A satisfactory rate performance can be obtained by increasing conductivity.^[^ ^86^, ^87^, ^88^, ^89^, ^90^ ^]^ Using high‐performance N‐doped carbon to modify conversion anode materials is an efficient approach to optimize their rate performance. Zhang et al. designed a composite anode material (Co_3_O_4_@N‐doped carbon) to improve the conductivity of metal oxide by the N‐doped carbon layer.^[^ ^91^ ^]^ The Co_3_O_4_@N‐doped carbon composite contain mainly three different types of nitrogen doping types (**Figure** **8**a). As shown in Figure 8c, due to the unpaired *p* electrons of pyridinic‐N increase in the electron cloud density of the overall π system, it has become more conductive through a significant shift from the valence band toward the conductive region. Therefore, the Co_3_O_4_@N‐doped carbon composite anode material has exhibited superior capacity (448.7 mAh g^−1^ at 0.05 A g^−1^) and satisfactory high‐rate performance (Figure 8b). Similarly, the tin (Sn) nanoparticles like cobalt oxide, suffer from low electrical conductivity. It can be addressed by combined with N‐doped carbon to enhance electrical conductivity. Li's group synthesized a novel mixed N‐doped carbon nanofiber framework (Sn/N‐CNFs). This material uniquely combines the benefits of porous Sn nanospheres made up of ultra‐small nanoparticles with a robust, interwoven N‐doped carbon nanofiber (N‐CNF) framework. The synergy between the ultra‐small nanoparticles and the distinctive carbon interconnection network significantly enhances conductivity and electron transfer capabilities. Consequently, Sn/N‐CNFs exhibit outstanding cycling performance, maintaining a capacity of 198.0 mAh g^−1^ at 1.0 A g^−1^ even after 3000 cycles, outperforming other Sn‐based materials reported at that time.^[^ ^92^ ^]^ Meanwhile, some researchers have also reported that the high capacity, and outstanding rate capability were attributed to the unique structure of metal oxide (porous, abundant defect sites, etc.).^[^ ^93^, ^94^, ^95^, ^96^ ^]^

The development of conductive networks is crucial for improving the electrical conductivity of composite materials. Wang et al. synthesized a high‐performance antimony selenide (Sb_2_Se_3_) anode through a combined strategy of conductive encapsulation and 2D confinement (Figure 8d).^[^ ^97^ ^]^ As shown in Figure 8e, the ratio of nitrogen doping components in the Sb_2_Se_3_@NC and Sb_2_Se_3_@NC@rGO composites. The introduction of rGO nanosheets leads to a significant reduction in the graphitic‐N component by 17.5%, while the pyridinic‐N and pyrrolic‐N components increase by 7.9% and 10.6%, respectively. An increase content of pyrrolic‐N and pyridinic‐N can significantly enhance the material's conductivity. The Sb_2_Se_3_ anode materials were encapsulated by a conductive carbon layer and confined within graphene nanosheets interlayers. These carbon coatings and graphene layers chemically integrate to enhancing overall conductivity. The conductive network formed by conductive carbon layer and confined within graphene nanosheets interlayers can effectively increase the rate capability. The rate capability of Sb_2_Se_3_@NC@rGO electrodes was assessed across current densities of 0.05, 0.1, 0.2, 0.5, and 1.0 A g^−1^, yielding capacities of ≈ 595, 545, 475, 270, 130 mAh g^−1^, respectively (Figure 8f). Notably, a continuous long‐term cycling test at 0.5 A g^−1^ after the rate capability test, the Sb_2_Se_3_@NC@rGO still showed a high capacity of 250 mAh g^−1^ over 350 cycles.

**Figure 9:**
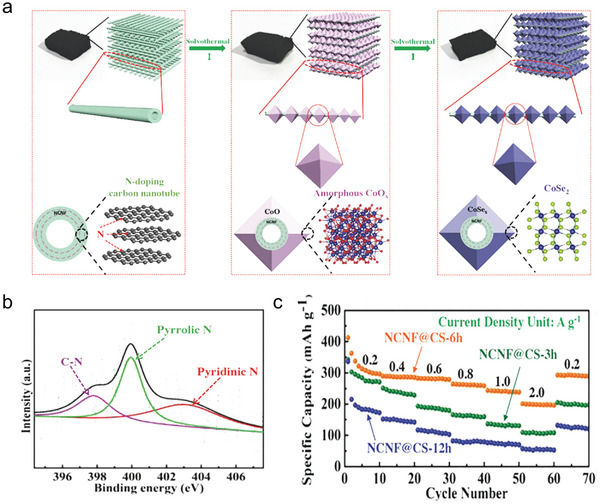
a) Schematic illustration of the synthesis process of NCNF, NCNF@CoO_x_, and NCNF@CS (From left to right). b) N 1s XPS spectrum. c) Rate performance under different current densities of the as‐prepared three NCNF@CS samples. Reproduced with permission.^[^ ^82^ ^]^ Copyright 2018, Wiley‐VCH.

Anchoring the material within a plane of N‐doped carbon further enhances electrical conductivity. Wu et al. developed a “gel‐blowing” method to confine FeS_2_ in an N‐doped carbon matrix.^[^ ^99^ ^]^ The external carbon shell on FeS_2_ nanoparticles inhibits aggregation and improves transfer kinetics. The as‐prepared FeS_2_@NC displayed a high specific capacity of 525.5 mAh g^−1^ at 0.1 A g^−1^ and a record long‐term cyclability with 0.016% capacity loss per cycle over 5000 cycles. When the current density was increased to high rates of 5 and 10 A g^−1^, the anode also showed excellent rate performance with capacities of 236 and 155 mAh g^−1^, respectively. This reported also systematically studied the reaction mechanisms during K^+^ storage, demonstrating a combination of intercalation and phase transformation. Simultaneously, N‐doped carbon materials providing a complex conductive network, significantly enhancing material conductivity. Yin et al. reported a metallic octahedral CoSe_2_ threaded by flexible framework N‐doped carbon nanotubes (NCNF@CS‐n, n is the reaction times) as anode material (**Figure** **9**a).^[^ ^84^ ^]^ According to the report, the special carbon nanotube framework structure can improve the electrical conductivity. The NCNF@CS exhibited the lowest resistance of 5.72 Ω when the reaction time was 6 h, which suggestion the NCNF@CS‐6 h occurred highest electrical conductivity. The highly conductive N‐doped carbon nanotubes can improve the rate performance by accelerates the electron transfer. As shown in Figure 9b, the composite N‐doped carbon materials contain three types of nitrogen doping: graphitic‐N, pyrrolic‐N (mainly), and pyridinic‐N. Owing to the high conductivity, the NCNF@CS‐6 h displays the rate performance under 0.2 to 2.0 A g^−1^. Reversible capacities of 297, 286, 279, 259, 238, and 196 mAh g^−1^ were observed after increasing the charge/discharge current densities to 0.2, 0.4, 0.6, 0.8, 1.0, and 2.0 A g^−1^, respectively (Figure 9c). Similarly, Liu et al. synthesized a FeSe_2_/nitrogen‐doped carbon (FeSe_2_/NC) composite with a structure of N‐doped carbon‐coated FeSe_2_ nanoparticles anchored on thin carbon nanosheets matrix as an anode material for PIBs.^[^ ^100^ ^]^ Benefiting from the nitrogen doping carbon coating buffer volume fluctuation and formed the complex conductive framework, the FeSe_2_/NC electrode delivers high capacities of 434 mAh g^−1^ after 70 cycles at 0.1 A g^−1^, and 301 mAh g^−1^ after 250 cycles at 1.0 A g^−1^. In summary, utilizing N‐doped carbon with high electrical conductivity to modify conversion anode materials effectively enhances their rate performance. First, the N‐doped carbons significantly boost carbon materials electrical conductivity through increasing the electron cloud density in the overall π system. Second, anchoring materials within the N‐doped carbon plane further enhances electrical conductivity. Lastly, N‐doped carbon can also create an intricate conductive network, significantly improving electrical conductivity (**Table** **3**).

**Table 3:** Summary of the advantages and disadvantages of nitrogen‐doped non‐carbonaceous materials synthesis strategies.

Non‐carbonaceous materials		Method strategies	Advantages	Disadvantages
Nitrogen‐doped carbon optimizes the conductivity	Co_3_O_4_@N‐doped carbon	Solvothermal synthesis	Cheap equipment; easy operation	The yield is limited by the container; uneven doping
	Sn/N‐CNFs	Electrospinning and thermal annealing	Easy operation; high efficiency	Uneven doping; high heat loss; exhaust gas pollution
	Sb_2_Se_3_@NC@rGO	Solvothermal synthesis	Cheap equipment; easy operation	Harsh feedstock; the yield is limited by the container; uneven doping
	FeS_2_@NC	Thermal annealing	Cheap equipment; easy operation; high efficiency; abundant feedstock	Less impurity, Oxygen species are easily introduced; low doping density
	NCNF@CoSe_2_	Chemical vapor deposition (CVD)	High efficiency; controllable doping ratio; large‐area preparation	Low yield; high temperature; exhaust gas pollution; complex operation
Nitrogen‐doped carbon buffers volume expansion	MSCNF@K	Electrospinning and thermal annealing	Mild conditions; easy operation	Uneven doping; high heat loss; exhaust gas pollution
	CoNC	Thermal annealing	Cheap equipment; easy operation; high efficiency; abundant feedstock	low doping density; uneven doping

### 4.2 Nitrogen‐Doped Carbon Buffers Volume Expansion of Anode Materials

The volume expansion during potassiation/depotassiation processes is inevitable for many high‐capacity anode materials. This volume expansion causes structural pulverization, leading to particle decohesion. Consequently, particle decohesion results in poor cycling stability. The porous structure serves as an effective strategy to optimize cycling stability. It ensures continuous contact between electrolyte ions and the anode material, while simultaneously mitigating the anode material's volume expansion. The N‐doped composite materials have attracted much attention because of its abundant porous structure. The N‐doped composite materials with porous structure have demonstrated an ability to effectively buffers volume expansion, thereby significantly enhancing cycling stability of anode materials. Standard methodologies for constructing pore structures include electrospinning techniques and self‐sacrificial template methods. These approaches facilitate the formation of diverse porous structures through mechanisms such as nanofibers’ electrospinning and templates’ self‐decomposition.

**Figure 10:**
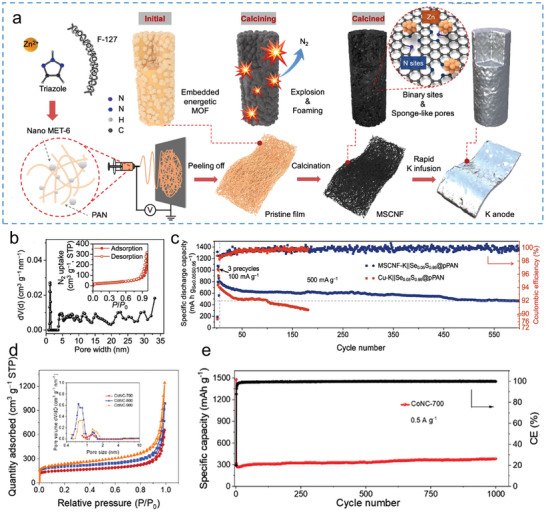
a) Synthetic route of MSCNFs and the corresponding composite K anode. b) Pore distribution of MSCNFs and the corresponding N_2_−77 K adsorption isotherms (inset). c) Long‐term cycling performance at 0.5 A g^−1^ of MSCNFs. a–c) Reproduced with permission.^[^ ^98^ ^]^ Copyright 2022, Nature Publishing Group. d) N_2_ adsorption‐desorption isotherm and pore size distribution of CoNC‐700, CoNC‐800, and CoNC‐900. e) Long‐term cycling performance of CoNC‐700 electrode at a current density of 0.5 A g^−1^. d,e) Reproduced with permission.^[^ ^99^ ^]^ Copyright 2022, American Chemical Society.

Potassium metal is one of the ideal anode materials for PIBs. The volume expansion of potassium metal anode during K plating/stripping greatly obstruction the development of potassium metal anode. Utilizing nitrogen‐doped carbon nanofibers to mitigate the volume expansion of potassium metal is an effective strategy to develop stable potassium metal anodes. Xie et al. reported nitrogen‐doped porous carbon nanofibers (the nanosized Zn‐triazole metal‐organic framework‐derived carbon nanofibers, MSCNFs) that act as potassium metal hosts through electrospinning and thermal treatment (**Figure** **10**a).^[^ ^97^ ^]^ The formation of pore structures was achieved through the evolution of gases from the triazole linkers with high nitrogen content when subjected to high‐temperature (600 °C) calcination. Figure 10b illustrates that the MSCNFs predominantly contain mesopores (>4 nm) and some micropores (1.2 nm), indicating a hierarchically porous structure. Benefitting from the hierarchical pores and the voids in the interconnected nanofibers, the MSCNFs can effectively buffer the volume expansion of potassium metal anode during K plating/stripping. The MSCNFs‐K, synthesized by compounding MSCNFs with potassium metal, demonstrate excellent cycling stability. As a result, the MSCNF‐K anode exhibited significantly enhanced stability, which can retain capacity ≈ 480 mAh g^−1^ over 600 cycles at 0.5 A g^−1^ (Figure 10c).

The self‐sacrificial template method is one of common technique for creating materials with intricate porous structures. By decomposing or removing templates during synthesis process, this method generates interconnected pores that enhance ion transport and accommodate volume changes during cycling. Liu et al. synthesized 3D N‐doped graphitic carbon spheres anchored with cobalt nanoparticles (CoNC) via self‐sacrifice template method.^[^ ^98^ ^]^ The microporous structure of CoNC not only provides enough space to handle the expansion of cobalt particles but also promotes the penetration of electrolytes into the anode material. The samples of CoNC‐700 (pore size, 1.33 nm), CoNC‐800 (pore size, 0.70 nm), and CoNC‐900 (pore size, 0.80 nm) were obtained by adjust the calcination temperature (Figure 10d). As the temperature increases, there is a corresponding reduction in pore size. The CoNC‐700 electrode with large microporous structure reaches 330 mAh g^−1^ at 0.5 A g^−1^ after 1000 cycles (Figure 10e) and 248 mAh g^−1^ at 2.0 A g^−1^ after 800 cycles. Accordingly, the N‐doped composite materials with a porous architecture have shown the capability to effectively mitigate volume expansion, which considerably improves the cycling stability of anode materials. First, in electrospinning technology, mesopores N‐doped composites can be prepared by calcining precursors with high nitrogen content. Then, the self‐sacrifice template method can also introduce pore structures through calcining template microspheres with nitrogen incorporation. Lastly, N‐doped composite materials with rich porous structures can effectively buffer the volume expansion of anode materials for PIBs.

## 5 Conclusion and Perspective

In summary, a fundamental understanding of the three different types of nitrogen in N‐doped carbon materials can help to promote the development of high‐performance anode materials for PIBs. In N‐doped carbon materials, pyridinic‐N can increase the electron cloud density of the overall π system to improve the electrical conductivity. Pyrrolic‐N can through electrostatic repulsion in the graphene layer to increases the interlayer spacing of carbon materials. Meanwhile, the pentagon‐ring and hexagon‐ring formed by pyrrolic‐N and pyridinic‐N can form the defective structure. These defects serve as active sites for electrochemical reactions. When the graphitic‐N content is reduced, the ICE can be improved. Lastly, the porous structure of N‐doped carbon materials can provide space to effectively buffer volume expansion of anode materials and ensure the electrolyte ions easily contact with the surface of electrode. Various synthetic methods have been developed to prepare N‐doped carbon materials. N‐doped carbon materials with a high nitrogen content can be prepared from the high N‐content precursors. Meanwhile, it is essential to note that nitrogen doping is beneficial for optimizing carbon materials for electrode materials, more doping is not always better. The optimal doping level needs to be carefully balanced to enhance electrical conductivity, reactivity, and structural stability without inducing undesirable effects. Despite promising progress in recent years, several key challenges are remained and more attentions should be paid to find possible solutions in the future. There are some perspectives on future research focuses in developing N‐doped carbon materials as PIB anode.

1. Different types of doped nitrogen have specific functional advantages. However, there is no suitable method to regulate the type and ratio of doped nitrogen. Future research should focus on refining nitrogen doping techniques to achieve precise control over nitrogen types and ratio within carbon materials. Employing advanced synthesis methodologies such as plasma‐enhanced chemical vapor deposition (PECVD), chemical vapor deposition (CVD) and electrochemical doping will enable more accurate regulation of doped nitrogen types and ratio. By optimizing these doping processes, it is possible to unlock new potentials for N‐doped carbon materials in PIBs.
2. The underlying mechanisms by the content of nitrogen doping influences capacity remain unclear. To achieve a comprehensive understanding of the content‐performance relationships in N‐doped carbon materials, it is essential to employ advanced characterization techniques (e.g., in situ extended X‐ray absorption fine structure (EXAFS), in situ transmission electron microscopy (TEM), etc.) and theoretical models (e.g., density functional theory (DFT), molecular dynamics (MD), etc.). It can better understand the interaction between nitrogen dopants and potassium ions at the atomic level. Future research should focus on exploring the synergistic effects between nitrogen dopants and other heteroatoms (e.g., sulfur, phosphorus, oxygen, boron, etc.) could provide additional avenues for enhancing capacity. Co‐doping strategies may modify the electronic environment further and facilitate enhanced ion adsorption and diffusion. Future research could focus on understanding the stability of nitrogen‐doped materials over prolonged cycling, identifying degradation pathways specific to different nitrogen contents, and developing strategies to mitigate these effects (e.g., through surface coatings or structural reinforcements). Additionally, combining machine learning with studying nitrogen doping mechanisms offers a promising approach to identifying optimal doping structures and quantities. Finally, combining theoretical modeling with experimental data can accelerate the discovery of highly efficient nitrogen‐doped anode materials. These strategies will not only elucidate the fundamental processes but also guide the rational design of N‐doped carbon materials with optimized electrochemical performance.
3. Developing large‐scale and low‐cost production methods can boost the commercialization of N‐doped carbon material. Meanwhile, green synthesis of N‐doped carbon materials will be an important development direction in the future. Research should focus on the development of environmentally sustainable synthesis technologies, such as the use of biomass as a raw material and the preparation of N‐doped carbon materials through low‐temperature, non‐toxic chemical processes, in order to reduce environmental pollution and resource waste.
4. The challenge in utilizing N‐doped carbon materials for PIBs lies in balancing the advantages and disadvantages of different nitrogen types. While pyridinic‐N and pyrrolic‐N improve adsorption capacity and provide more active sites, they also introduce defects and decrease conductivity. In contrast, graphitic‐N maintains structural stability and high conductivity but has limited reactivity. Future research should aim to optimize the proportion and distribution of various nitrogen types to maximize the overall electrochemical performance while minimizing the disadvantages, such as structural disruption and reduced ICE.

In the future, N‐doped carbon materials will open the way for achieving high‐performance PIBs and offer new possibilities for enhancing the electrochemical performance of carbon materials in terms of K^+^ storage.

## Conflict of Interest

The authors declare no conflict of interest.
